# Methodological considerations for the identification of choline and carnitine-degrading bacteria in the gut

**DOI:** 10.1016/j.ymeth.2018.03.012

**Published:** 2018-10-01

**Authors:** Eleanor Jameson, Mussa Quareshy, Yin Chen

**Affiliations:** The University of Warwick, School of Life Sciences, United Kingdom

## Abstract

The bacterial formation of trimethylamine (TMA) has been linked to cardiovascular disease. This review focuses on the methods employed to investigate the identity of the bacteria responsible for the formation of TMA from dietary choline and carnitine in the human gut. Recent studies have revealed the metabolic pathways responsible for bacterial TMA production, primarily the anaerobic glycyl radical-containing, choline-TMA lyase, CutC and the aerobic carnitine monooxygenase, CntA. Identification of these enzymes has enabled bioinformatics approaches to screen both human-associated bacterial isolate genomes and whole gut metagenomes to determine which bacteria are responsible for TMA formation in the human gut. We centre on several key methodological aspects for identifying the TMA-producing bacteria and report how these pathways can be identified in human gut microbiota through bioinformatics analysis of available bacterial genomes and gut metagenomes.

## Highlights

• Bacterial pathways have previously been elucidated for trimethylamine formation.
• We explain how to identify human-associated bacteria that produce trimethylamine.
• How to identify which gut bacteria are responsible for producing trimethylamine.
• Are we missing anything – unknown bacterial trimethylamine formation pathways?

## 1 Introduction

The human gastro-intestinal tract is an ecosystem rich in microbial diversity. Gut microbiota encompasses diverse groups of bacteria, archaea, viruses, fungi and other microeukaryotes [1], [2], [3], [4], [5]. It has been estimated that, on average, 3.8 × 10^13^ bacteria inhabit the human gut, which encompasses over 1000 species [6], [7]. It is now evident that gut microbiota have an important role in human health and disease. In a healthy gut, these microbes form a stable community whereas during gut dysbiosis, opportunistic pathogens and parasites thrive [8]. Understanding the complex interactions and metabolic capacity of the gut microbiome will help us to examine the workings of this microbiome and better manage disease.

Recent work on the role of the human gut microbiome in disease has linked the bacterial metabolite trimethylamine (TMA) with atherosclerotic cardiovascular disease (ACVD). Previous studies on humans and experimental animals have indicated that choline and carnitine, both of which are conditional B-type vitamins, are the major dietary precursors of TMA in the gut [9], [10], [11], [12], [13], [14], [15], [16], [17]. TMA formation from choline and carnitine is linked to ACVD through hepatic formation of trimethylamine *N*-oxide (TMAO) although the underlying molecular and cellular mechanisms remain to be fully established [9], [15], [18].

The metabolic pathways responsible for bacterial transformation of choline and carnitine to TMA were unknown until very recently [10], [17]. It is interesting that choline and carnitine degradation to TMA involves some unique chemistry – the former requires a glycyl radical, encoded in the choline-TMA lyase, CutC protein for the breakage of the carbon-nitrogen bond, whereas the latter employs a mononuclear iron in the active centre in the CntA protein. CutC belongs to a larger family of enzymes sharing the same glycyl radical chemistry and shows significant sequence homology to other members of the family such as glycerol dehydratase and pyruvate formate lyase [10], [19]. Similarly, CntA is a new member of the Rieske-containing oxygenases and has significant sequence homology to several members of the Rieske protein family [17]. Although the enzymes responsible for TMA formation from these compounds have now been established, identifying of the microbiota species in the human gut that are responsible for TMA formation remains a challenging task. We focus on several key methodological aspects, which require particular attention when identifying the TMA-producing bacteria and report how to identify these TMA-production pathways in the human gut microbiome through analyses of published bacterial genomes and microbiomes.

## 2 Pathways of TMA production by gut microbiota

**Fig. 1:**
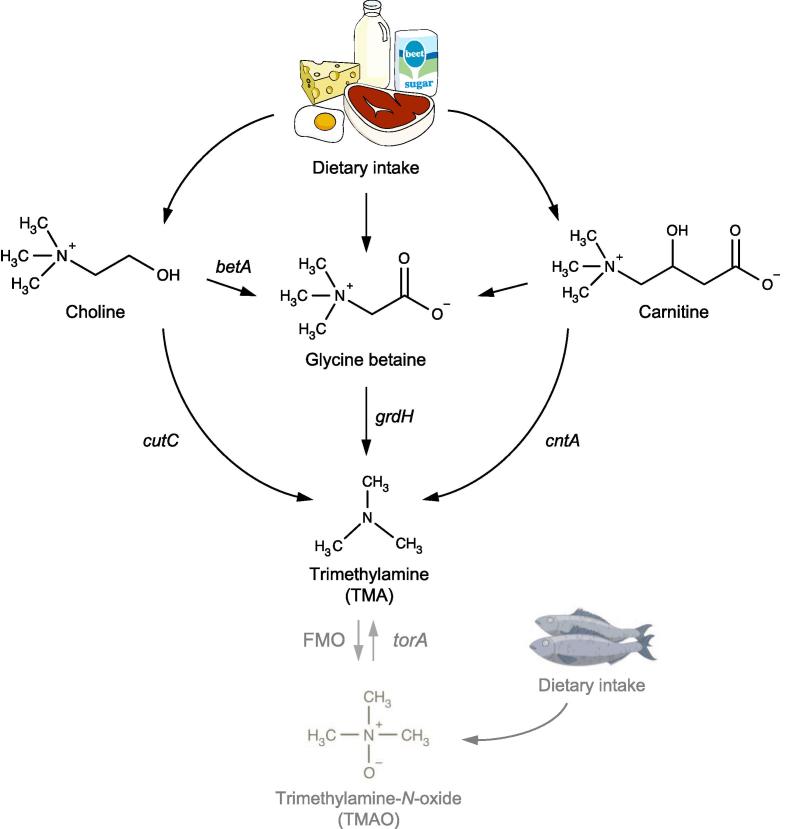
Known pathways for the formation of trimethylamine (TMA) from dietary choline and carnitine. The key enzymes responsible for TMA production indicated in the figure are: CntAB, carnitine monooxygenase [17] which is analogous to YeaW; CutCD, choline-TMA lyase [10] and GrdH, glycine betaine reductase [20]. Choline to glycine betaine is mediated by the Bet pathway [48], a pathway from carnitine to glycine betaine has been proposed, but has yet to be elucidated [49], [50]. Additionally shown in dark grey the TorA, trimethylamine N-oxide reductase [21] and TMAO formation pathway FMO, flavin-containing monooxygenase are critical to TMA cycling, but are not the focus of this review [51], [52].

Several pathways for bacterial TMA formation are currently known (Fig. 1), involving a choline-TMA lyase, CutC [10], [19], a carnitine monooxygenase, CntAB [17], a glycine betaine reductase, GrdH [20], or additionally via the reduction of TMAO [21], [22], [23]. Here we focus on the production of TMA from choline and carnitine, since they have been shown to be relevant in cardiovascular disease [9], [24]. These two pathways have been determined using a combination of comparative omics, bioinformatics, molecular genetics and biochemistry based on several model laboratory bacterial strains [10], [17], [19].

The enzyme that catalyses the degradation of choline to TMA under anaerobic conditions has been identified as the glycyl radical-containing enzyme CutC [10], [19]. Interestingly, the *cut* gene cluster also houses a set of genes encoding a microcompartment [10], [19]. Although the *cut* gene cluster was originally characterized from a sulfate-reducing deltaproteobacterium, *Desulfovibrio desulfuricans* [10], subsequent analyses have shown that *cutC* homologues and the shell proteins involved in microcompartment formation also occurred in gut *Gammaproteobacteria*, *Actinobacteria* and *Firmicutes* [12], [19], [25]. The formation of a functional microcompartment in choline metabolism has been experimentally demonstrated in *Proteus mirabilis* [19].

**Fig. 2:**
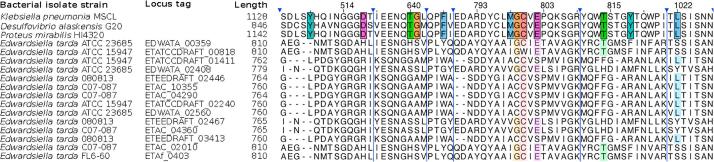
A sequence alignment of characterized CutC proteins from *Klebsiella pneumonia*[34], *Desulfovibrio alaskensis*[35] and *Proteus mirabilis*[19] aligned with Clustal Omega [53] to blast matching genes from *Edwardsiella tarda*. Despite showing a global high matching sequence similarity, when we compare the key binding site residues (coloured), we observe very little concensus. Sequences are visualised in JalView [54].

Further to the Cut pathway, it has been previously hypothesized that other pathways capable of degrading choline to TMA may also exist, as exemplified by *Edwardsiella tarda* ATCC 23685. It has been demonstrated that although this bacterium was capable of producing TMA from choline, it appeared lacking the characterized *cut* gene cluster [14]. A close examination of the recently published genomes of *Edwardsiella tarda* has revealed the presence of several glycyl radical family proteins (*Edwardsiella tarda* ATCC 23685 EDWATA_00359: E-value 9e-136; identity 33% to characterized CutC of *P. mirabilis* [19]), which are annotated as pyruvate-formate lyases (EC: 2.3.1.54) (Fig. 2). However the key choline-binding site residues showed very low consensus to confirmed CutC proteins (Fig. 2). It therefore remains to be tested whether these so-called pyruvated-formate lyases are functional choline-TMA lyases, or indeed a novel glycyl radical-independent pathway exists in *Edwardsiella tarda* for choline degradation to TMA.

**Fig. 3:**
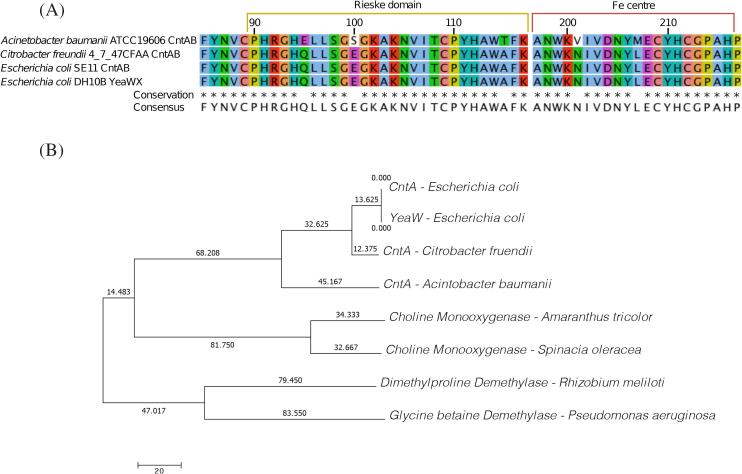
A. Protein alignment of CntA and YeaW showing sequence conservation of the functional domains based on representative CntA sequences from functional confirmed *Citrobacter freundii* CntA, *Acinetobater baumanii* CntA, *Escherichia coli* CntA and Escherichia coli YeaW. The alignment shows a high level on sequence conservation between CntA sequences and the functional domain of YeaW is indistinguishable. B. Phylogenetic tree of Rieske oxygenases based on full length protein sequences with bootstrap values. A. and B. combined show that the CntA from *Escherichia coli* SE11 [17] and YeaW from *Escherichia coli* DH10B [26], [27] have an identical protein sequence. CntA of *Citrobacter freundii* and *Acinetobater baumanii* cluster with the other CntA proteins and are separate from other Rieske oxygenases used as outgroups.

An aerobic pathway for the degradation of carnitine to TMA has been identified by our groups using *Acinetobacter baumannii* as a model. The enzyme, carnitine monooxygenase encoded by the *cntA* gene, represents a novel protein of the Rieske oxygenase family, which is best known for several enzymes involved in the oxidation of aromatic compounds [17]. In *Escherichia coli* and closely related bacteria, CntA is sometime also referred as YeaW in the literature [26]. YeaW is sometimes described as a novel enzyme targeting γ-butyrobetaine [27], however, we and others have shown that *E. coli* YeaW is homologous to the CntA of *A. baumannii* through phylogenetic analysis, PFAM domain analyses and substrate specificity tests (Fig. 3) [28], [29], [30]. The CntA enzyme has been identified in several key gut microbiota groups, including *Proteobacteria*, *Actinobacteria* and *Firmicutes* [17], [27], [31], [32].

## 3 Identification of TMA formation potential in bacterial isolates

**Fig. 4:**
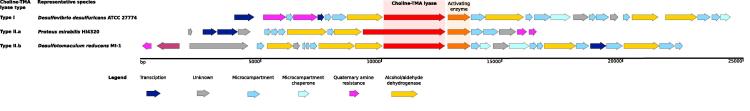
Alignment of the *cut* gene clusters for bacterial isolates representative of the three choline-TMA lyase (*cutC*) types [19]. The clusters are aligned to the homologous regions of the glycyl radical enzyme (GRE), *cutC* and activating enzyme, *cutD*. Gene functions are denoted by colour and the legend shown at the bottom.

**Fig. 5:**
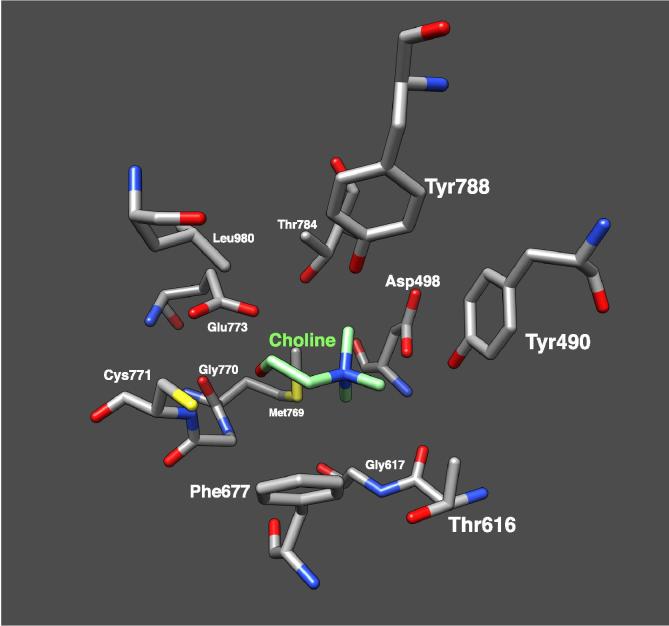
The active site of CutC choline-trimethylamine lyase from *Klebsiella Pneumoniae* (PDB 5A0U) showing the key residues involved in substrate recognition and enzymatic activity, visualised in Chimera [55].

One of the most widely-used and simplest approaches to identify gut-associated microbes with the potential to metabolise choline and carnitine to TMA is *in silico* BLAST searches of the key signature genes against microbial genome databases such as IMG (https://img.jgi.doe.gov/cgi-bin/m/main.cgi) and NCBI [12], [29]. BLAST searches are carried out using single amino acid sequences of a key gene (with a proven function, Table 1) as the query and comparing this, by means of local alignments, to all of the sequences in a given database. The BLAST searches result in hits to similar gene sequences and provides measures of their statistical significance and can be carried out directly through databases sites such as IMG and NCBI. Using key genes from the choline (*cutC*) and carnitine (*cntA*) to TMA pathways as BLAST search queries, a number of hits, or gene homologues, can be identified in fully sequenced human-associated bacterial genomes. However, caution must be used when using this approach. It has been well-documented that the presence of a *cutC* homologue alone is insufficient to predict choline usage, due to a number of closely related genes with different substrate specificity, even within a single bacterial genome [12], [14], [19], [29], [32]. The gene synteny of the *cut* cluster provides a genetic context [12], the presence of the activator enzyme gene (*cutD*) and microcompartment genes, in addition to *cutC*, may be a better predictor of function in genome sequenced bacteria than *cutC* alone (Fig. 4). Again, this approach is not without pitfalls. It has been documented that several microbial species of the *Pelobacter* genus can metabolism choline to TMA [33], [34] and yet the genomes do not appear to contain microcompartment genes in the neighborhood of the *cutC/D* genes. Alternatively the protein structure is available for CutC (Fig. 5), which would help to determine the active site, enabling the validation of choline-binding CutC (Fig. 2) [35], [36].

**Table 1:** Bacterial strains confirmed to degrade choline or carnitine to TMA and containing either the *cutCD* or *cntAB* pathways respectively.

	Organism	Reference	Choline	Carnitine
Proteobacteria	*Acinetobacter baumannii* (ATCC 19606)	Zhu 2014 [17]		+
	*Acinetobacter calcoaceticus* (ATCC 39647)	Ditullio 1994 [56]		+
	*Acinetobacter calcoaceticus* 69/V	Kleber 1977 [57]		+
	*Citrobacter freundii* 4_7_47CFAA			+
	*Desulfovibrio alaskensis* G20	Weimer 1988 [58]	+	
	*Desulfovibrio desulfuricans* (ATCC 27774)	Craciun 2012 [10]	+	
	*Desulfovibrio desulfuricans* subsp. aestuarii (DSM 17919)	Rath 2017 [29]	+	
	*Escherichia coli* BL21-DE3	Kalnins 2018 [28]		+
	*Escherichia coli* DH10b	Koeth 2014 [24]		+
	*Escherichia coli* K12 (DSM 10517)	Rath 2017 [29]		+
	*Escherichia coli* MS 200-1	Romano 2017 [59]	+	
	*Escherichia coli* MS 69-1	Campo 2015 [12]	+	
	*Escherichia coli* SE11	Zhu 2014 [17]		+
	*Escherichia fergusonii* (ATCC 35469)	Romano 2015 [14]	+	
	*Klebsiella pneumoniae* (MSCL 535)	Kuka 2014 [16]/Kalnins 2018 [28]		+
	*Klebsiella pneumoniae* (MSCL)	Kalnins 2015 [35]	+	
	*Klebsiella* sp. MS 92-3	Campo 2015 [12]	+	
	*Pelobacter acetylenicus*	Schink 1985 [34]	+	
	*Pelobacter carbinolicus*	Aklujkar 2012 [33]	+	
	*Proteus mirabilis* (ATCC 29906)	Campo 2015 [12]	+	
	*Proteus mirabilis* (DSM 4479)	Jameson 2016 [19]	+	
	*Proteus mirabilis* BB2000	Campo 2015 [12]	+	
	*Proteus mirabilis* HI4320	Campo 2015 [12]	+	
	*Proteus penneri* (ATCC 35198)	Romano 2015 [14]	+	
	*Proteus vulgaris*	Seim 1982 [60]	+	
	*Providencia rettgeri* (DSM 1131)	Romano 2015 [14]	+	
	*Providencia rettgeri* (MSCL 730)	Kalnins 2018 [28]		+
	*Serratia marcescens* (MSCL 1476)	Unemoto 1966 [61]/Kalnins 2018 [28]		+
	*Vibrio cholinicus*	Hayward 1959 [62]	+	

Firmicutes	*Anaerococcus hydrogenalis* (DSM 7454)	Romano 2015 [14]	+	
	*Clostridium asparagiforme* (DSM 15981)	Romano 2015 [14]	+	
	*Clostridium citroniae* WAL-17108	Campo 2015 [12]	+	
	*Clostridium hathewayi* (DSM 13479)	Rath 2017 [29]	+	
	*Clostridium hathewayi* (DSM 13749)	Romano 2015 [14]	+	
	*Clostridium sporogenes* (ATCC 15579)	Romano 2015 [14]	+	
	*Streptococcus dysgalactiae* (ATCC 12394)	Campo 2015 [12]	+	

Actinobacteria	*Olesnella uli* (DSM 7084)	Campo 2015 [12]	+

It is important to note that such homology based phylogenetic analysis of *cutC* and *cntA* in bacterial isolates takes us one step away from the functionally verified genes and inevitably introduces margins for errors. The identified homologues may or may not encode a functional enzyme and additional experiments (e.g. by heterologous expression in a non-TMA producer) are required to validate the functionality of CutC/CntA proteins in TMA formation [10], [17], [19]. Wherever it is possible to obtain the bacterial isolate, the bacterium’s ability to produce TMA from choline should be experimentally tested [10], [17].

## 4 Identification of TMA formation potential in microbiome studies

Recent studies have begun to resolve which microbial species are responsible for TMA formation and degradation in the human gut through analysis of metagenomic datasets [12], [29], [31], [32], [37], [38]. This approach has involved analysing existing human gut metagenomes in search of key TMA metabolic genes [31], [32], screening fecal samples for the presence of these key genes [12], [29] or sequencing the faecal microbiomes of patients suffering with atherosclerotic cardio vascular disease [37].

To screen existing human gut metagenomes for TMA-production pathways and TMA-degradation pathways, metagenome data can be retrieved from public repositories, such as MG-RAST (http://metagenomics.anl.gov/) or specific metagenomes, such as the Karlsson data set (http://sra.dnanexus. com/studies/SRP016067) [29], [31], [32], [38]. Alternatively, Jie et al. [37] undertook targeted clinical sample collection, focusing on the fecal metagenome of ACVD patients to link the gut microbiome and TMA-production pathways with ACVD [39]. The primary methods utilised to identify TMA-production pathways from metagenome data are BLAST and Profile Hidden Markov Models (profile-HMM) [29], [31], [32]. The basis of BLAST (see Section 3) is a single representative amino acid sequence, while profile-HMMs, adapted originally from speech recognition algorithms, use multiple aligned amino acid sequences that are representative of a key enzyme or a specific protein family. The profile-HMMs rely on probabilistic models and take into account key amino acids and gaps, rather than identifying the percentage match of an entire sequence, as BLAST does. The two alternate methods have different advantages, BLAST only requires a single confirmed sequence, while profile-HMMs are based on the conserved motifs within a functional protein family, which reduces the bias innate to the BLAST approach [40]. BLAST and profile-HMM searches can be carried out locally using BLAST+ (NCBI) and HMMER (hmmer.org), respectively. After generating BLAST or profile-HMM hits it is crucial to reduce false positive hits, to homologues with unrelated functions, which can account for >90% of the total hits. This can be achieved through phylogenetic comparison of the hits to reference strains with known functions [31]. Likewise false negatives can further confuse results, e.g. assigning *cutC* homologues as pyruvate-formate lyase may account for results of Jie et al. [37], who report that ACVD patients had a significantly higher incidence of *cntA* than *cutC* (contrary to previous findings [29], [31], [32]), and a significant enrichment of pyruvate-formate lyase homologues. Wherever possible, one should 1) examine the key signature residuces of metagenome-derived CutC/CntA for substrate co-ordinnation; 2) confirm the function of metagenome-derived CutC/CntA by over-expression in a foreign hosts; and 3) obtain the (most closely related) bacterial isolate and test the bacterium’s ability to produce TMA [10], [17].

An alternative approach to using metagenome data is to utilise the abundant 16S rRNA gene sequencing data available and infer microbiome function, i.e. TMA degradation, based on taxonomy. There are various programs available, such as PICRUSt [41], Tax4Fun [42], Piphillin [43] and Vikodak [44] that aim to predict function from 16S rRNA gene sequences. These allow the user to get an idea of the potential metabolic function of a microbial community. The function prediction programs rely on how accurate and comprehensive the databases are, fortunately for studying the human gut, these programs have been developed using the human microbiome project and gut microbiome databases are sufficiently comprehensive, resulting in 75–85% average correlations to metagenome data, at predicting community level functions [3], [41], [42], [43], [44]. Another consideration for specific functions is how evenly distributed the functions of interest are across bacterial families or genera. Matching 16S rRNA gene taxonomy and metabolic function can be difficult to reconcile for some functions, due to gene loss between closely related strains and lateral gene transfer [45], [46]. The carnitine degradation genes *cntAB* have been detected in *Proteobacteria,* while the choline degradation *cutCD* genes have been detected in *Proteobacteria*, *Firmicutes* and *Actinobacteria* (Table 1), however these functional genes are unevenly distributed between bacterial phyla preventing accurate prediction of function through 16S rRNA genes alone [10], [12], [32]. PICRUSt has been applied to predict significant differences in genes involved in choline, carnitine and trimethylamine (TMA) metabolism in chronic kidney disease gut microbiomes [47], however these predictions should inform, rather than replace shotgun metagenome sequencing for accurate analysis of metabolic functions in a community.

## 5 Summary

In the rapidly developing and expanding field of gut microbiome-associated disease, microbial approaches are vital to understanding the causes of disease and the complex community interactions. The identification of metabolic pathways responsible for TMA production has informed molecular and clinical approaches that identify the species responsible for generating TMA in the human gut. A more in-depth understanding of these processes and an exhaustive identification of all bacterial TMA-production pathways will help to complete the picture of gut microbiome structure and function in terms of TMA-related disease pathogenesis. Direct links between diet, bacterial communities and human disease have been discovered, through a combination of molecular microbial approaches, these tools pave the way for future studies to expand the field of human microbiome caused disease.
